# Sitagliptin Stimulates Endothelial Progenitor Cells to Induce Endothelialization in Aneurysm Necks Through the SDF-1/CXCR4/NRF2 Signaling Pathway

**DOI:** 10.3389/fendo.2019.00823

**Published:** 2019-11-26

**Authors:** Guo Yu, Peixi Liu, Yuan Shi, Sichen Li, Yingjun Liu, Wei Zhu

**Affiliations:** ^1^Department of Neurosurgery, Huashan Hospital of Fudan University, Shanghai, China; ^2^Neurosurgery Institute of Fudan University, Shanghai, China

**Keywords:** sitagliptin, endothelial progenitor cells, aneurysm, SDF-1, CXCR4, NRF2

## Abstract

Aneurysm (AN) embolization is an important treatment for cerebral aneurysms. The endothelialization of the aneurysm neck is crucial for preventing aneurysm recurrence. Sitagliptin is a therapeutic drug for diabetes that has been reported to have cardiovascular-protective effects. This study investigated the effect of sitagliptin on endothelial progenitor cell (EPC) function and endothelialization of aneurysm necks after embolization. The effect of sitagliptin on aneurysm neck endothelialization was examined using a rat aneurysm embolization model. We isolated EPCs and used CCK-8 (Cell Counting Kit-8) and annexin V/PI to analyze the effect of sitagliptin on the proliferation and apoptosis of EPCs. The effects of sitagliptin on the migration and invasion of EPCs were examined using scratch and Transwell assays. The effect of sitagliptin on the angiogenic ability of EPCs was examined using a sprouting assay. Western blot analysis and ELISA were used to analyze the effect of sitagliptin on the expression of proangiogenic factors in EPCs. The *in vivo* results indicated that sitagliptin promoted endothelialization of the aneurysm neck and increased circulating EPCs and expression levels of SDF-1 and VEGF in peripheral blood. Sitagliptin promoted the proliferation, migration, invasion, and angiogenic abilities of EPCs. Western blot analysis and ELISA showed that sitagliptin promoted the expression of SDF-1 and VEGF in progenitor endothelial cells. Western blot assays showed that sitagliptin activated the expression of NRF2, which is dependent on the function of CXCR4. Furthermore, sitagliptin promoted progenitor endothelial cell migration, invasion and angiogenesis through the SDF-1/CXCR4/NRF2 signaling pathway. Additionally, progenitor endothelial cells expressed SDF-1 and VEGF. The promotion of endothelialization by sitagliptin provides an additional therapeutic option for preventing the recurrence of AN.

## Introduction

Intracranial aneurysms (IAs) are one of the main causes of subarachnoid hemorrhage due to the abnormal expansion of the arterial wall and occur due to various reasons (1). The global incidence of intracranial aneurysm is one in 10,000 people and is most common in people aged 40–66 years (1). Subarachnoid hemorrhage caused by rupture of aneurysms occurs suddenly and severely, which is an important cause of human death. Related studies have suggested that disorders of endothelial cell function, loss of parietal cells, infiltration of inflammatory cells, and degradation of extracellular matrix participate in the formation of aneurysms (2, 3). Currently, coil embolization is a relatively safe, effective, rapid, economical, and less invasive method for treating aneurysms compared with clipping therapy (4). However, the long-term recurrence rate is as high as 24% (5), and so preventing the long-term recurrence of cerebral aneurysms after embolization is the most noteworthy issue in interventional therapy. In recent years, an increasing number of studies have focused on the process of endothelialization of the aneurysmal neck after embolization, which prevents the recurrence of cerebral aneurysms to a certain extent (5). Endothelialization of the aneurysmal neck after coil embolization of intracranial aneurysms is essential for the prognosis of intracranial aneurysms (5). Incomplete endothelialization of intracranial aneurysms increases the risk of recurrence (6). Therefore, promoting the degree of endothelialization of intracranial aneurysms could help improve the prognosis of patients.

In 1997, Asahara et al. first discovered that endothelial progenitor cells are present in peripheral blood, which gradually became a popular topic of research (7). Endothelial progenitor cells are precursor cells of endothelial cells and are pluripotent stem cells (8, 9). Endothelial dysfunction is an early pathophysiological change in the formation of intracranial aneurysms and is the starting point and triggering factor for the formation of intracranial aneurysms (10, 11). EPC-mediated therapy has been proposed as a potential treatment for vascular disease. The SDF-1/CXCR4 pathway plays an important role in endothelial formation, angiogenesis, and hematopoiesis (12). Studies have shown that SDF-1 promotes the expression of VEGF in endothelial cells and is an important cytokine for mobilizing EPCs (13). SDF-1 is a CD34+ hematopoietic progenitor chemokine that mediates the migration and homing of hematopoietic progenitor cells. Studies on SDF-1 knockout mice have shown that SDF-1/CXCR4 plays an important role in cardiovascular function (14, 15). Nuclear factor-erythroid 2-related factor 2 (Nrf2), a protein that was discovered by Moil et al. in 1994, is a bZip (leucine zipper) transcription factor (16). Previous studies have shown that Nrf2 has a protective effect on the cardiovascular system, such as protecting cardiomyocytes from ischemia-reperfusion injury. Recent studies have shown that Nrf2 exerts an effect in the process of angiogenesis and that Nrf2-related pathways play a key role in the proliferation of hematopoietic stem cells and the maintenance of stem cell characteristics, and Nrf2 can directly bind to the promoter of CXCR4 to regulate the expression of CXCR4 (17).

As an oral antidiabetic agent, sitagliptin is commonly used as a monotherapy or in combination with other oral hypoglycemic agents for the treatment of T2DM by inhibiting dipeptidyl peptidase IV (DPP IV) activity. The pharmacological effect of DPP-4 inhibitors is to prolong the action of incretin hormones and avoid degradation of SDF-1 (18). Previous studies have shown that sitagliptin affects vascular repair and remodeling (19, 20).

Therefore, this study aimed to verify whether sitagliptin can mobilize EPCs to promote the endothelialization of aneurysms, prevent recurrence after aneurysm embolization, and provide ideas for future drug treatment after aneurysm embolization. However, the effect of sitagliptin on promoting EPCs to stimulate angiogenesis and vascular function is largely unknown. Because of the importance of endothelialization of aneurysms after embolization, we investigated the effect of sitagliptin on EPC mobilization and the active treatment of intracranial aneurysms.

## Materials and Methods

### Rat Coiled Aneurysm Model

The establishment of a rat coiled aneurysm model has been described in previous studies (4). The animal experiments involved in this study were strictly in accordance with the requirements of the Ethics Committee of Fudan University and were approved by the Ethics Committee of Fudan University. Briefly, a blood vessel from a healthy rat (Sprague-Dawley, male, 8 weeks old, 200–250 g) abdominal aorta was used, and an anastomotic aneurysm occurred between the abdominal aorta, renal vein, and iliac vein. The proximal region of the aneurysm was confined by partial ligation to create an aneurysm neck, and the distal region of the anastomotic segment was completely ligated. A coiled aneurysm model was created via Guglielmi detachable coil (GDC) embolization prior to distal ligation. The animals were randomly divided into four groups: (1) Animals in the mock-surgery group (MS group, *n* = 12) (2) Animals in the aneurysm treated without any intervention (AN group, *n* = 12) (3) Animals in the aneurysm treated with sitagliptin via gavage (AN+ sitagliptin group, *n* = 12), and (4) Animals in the aneurysm treated with sitagliptin combined with AMD3100 by intraperitoneal injection group (AN+ sitagliptin + AMD3100 group, *n* = 12). Sitagliptin (Merck, # Y0001812) was administered gavage at 15 mg/kg/day for 30 consecutive days, and the combined treatment group also received AMD3100 (Merck, #239820) (A symmetrical bicyclam compound that antagonizes CXCL12 (SDF-1) binding to CXCR4) intraperitoneally at 1.25 mg/kg/day for 30 consecutive days.

### Scanning Electron Microscopy and H&E Staining

For scanning electron microscopy (SEM), the aneurysm neck specimen was gently removed after 30 days, fixed with 0.25% glutaraldehyde overnight, and then dehydrated. Scanning electron microscopy was performed after specimen dehydration was completed. For H&E staining, the aneurysm specimen was placed in 4% PFA (paraformaldehyde) for fixation and then subjected to dehydration treatment. After dehydration, waxing, and paraffin embedding were carried out, paraffin sectioning of the aneurysm neck was performed at a thickness of 7 μm, and H&E (Hematoxylin and Eosin) staining was then performed.

### Immunofluorescence

Thirty days after the establishment of the rat coiled aneurysm model, three groups of aneurysms were removed, and paraffin sections of the aneurysmal neck were stained to observe endothelial fluorescence. Incubation with primary anti-vWF (Von Willebrand Factor) (Abcam, #6994) or CD31 antibodies (Abcam, #182981) was performed overnight at 4°C. The primary antibodies were detected using FITC-conjugated anti-rabbit secondary antibodies (ThermoFisher, #A32731). After the final wash, the nuclei were counterstained by adding 2 mg/ml DAPI (ThermoFisher, #D1306) in PBS for 10 min before imaging. Cells were visualized using a confocal microscope.

### Isolation and Identification of EPCs

This study was approved by the Institutional Animal Care and Use Committee and the Ethics Committee of Fudan University. Eight-week-old male Sprague-Dawley rats were used for EPC isolation. Bone marrow was isolated from the femur and tibia of the rats and then subjected to density gradient centrifugation using Ficoll (Sigma-Aldrich, #10771). Then, mononuclear cells were cultured on collagen I-coated dishes with EGM-2 medium (Lonza, #CC-3202) at 37°C in a 5% CO_2_ incubator. After 3 days of incubation, the medium was changed every 3 days. When the cells had reached the second generation, the cells were stained with Dil (Sigma-Aldrich, #42364) and UEA (Sigma-Aldrich, #L9006). Laser confocal microscopy was used for observation of Dil and UEA staining.

The mononuclear cells in the peripheral blood were isolated by Ficoll density gradient centrifugation at 10, 20, or 30 days after the aneurysm model was established. CD34 (Abcam, #ab81289) and KDR (Vascular endothelial growth factor receptor-2, VEGFR-2) (Abcam, #ab9530) monoclonal antibodies were incubated with the mononuclear cells for 1 h on ice and then incubated with Alexa Fluor 546- (ThermoFisher, #A10036) and Alexa Fluor 488-(ThermoFisher, #A11008) labeled fluorescent secondary antibodies for 30 min. The proportion of CD34+/KDR+ cells was analyzed by flow cytometry (Beckman Coulter, DxFLEX).

### Cell Viability Assay With CCK-8 and Flow Cytometry

EPCs were seeded onto 96-well plates at 1 × 10^4^ cells per well. After adding different concentrations of sitagliptin, 10 μl of CCK-8 solution was added at different timepoints and incubated at 37°C for 2 h. Then, the absorbance values were analyzed by a microplate reader at a wavelength of 450 nm. The apoptotic rate of the EPCs was analyzed by using an annexin V/PI kit (ThermoFisher, #V13245) according to the manufacturer's instructions. The cells were suspended in binding buffer and stained with 5 μl of annexin V and 10 μl PI for 15 min at room temperature (RT) in the dark. Cells were excited at 488 nm, and the signals from 10,000 cells were acquired. The results were analyzed using FACStar (BD Biosciences).

### EPC Migration and Invasion Assays

For the scratch assay, cells were seeded onto six-well-plates and scratched with a pipette tip after the cells had grown to full confluence. Then, the medium was replaced with serum-free medium, different concentrations of sitagliptin were added, and the cells were observed after 24 h. For the Transwell assay, 200 μl of serum-free medium containing 2 × 10^4^ EPCs were seeded into the upper chamber with different concentrations of sitagliptin, and 600 μl of EBM-2 medium containing 1% serum was added to the lower chamber. After 24 h of culture in a cell culture incubator, the cells in the upper chamber that did not migrate were removed using cotton swabs, fixed with 4% PFA for 15 min, and then stained with 1% crystal violet (Sigma-Aldrich, #C0775) for 30 min.

### Tube Formation Assay

The tube formation assay is a rapid and quantifiable method for measuring vascular formation *in vitro*. EPCs are bound to conditioned media and inoculated into basement membrane extracts. It is easy to quantify the newly formed tubules within hours of tube formation. For the tube formation assay, EPCs were pretreated with sitagliptin for 24 h. The individual wells of a 96-well-plate were coated with 30 μl. Matrigel basement membrane matrix (BD Biosciences). Next, 3 × 10^5^ sitagliptin-treated EPCs were gently added to each gel-coated well. After 20 h, the cells were dyed with calcein AM and examined via fluorescence microscopy. Images were acquired and analyzed for tube formation using the Wimasis image analysis program. The total number of nets (distinct regions of tubes that contain at least 1 branching point) was used to estimate the level of tube formation. Isolated tubes were not considered nets.

### Real-Time PCR

Total RNA from EPCs was extracted using TRIzol reagent (ThermoFisher, #15596018) according to the manufacturer's instructions. mRNA was reverse transcribed according to the manufacturer's instructions using a reverse transcription kit (Takara, RR036A). PCR was carried out according to the manufacturer's instructions using a SYBR^®^ Premix Ex Taq^TM^ II Kit (Takara, RR420A). The relative expression value of a target gene was calculated according to the 2^−ΔΔCt^ method. The primer sequences were as follows: SDF-1: forward primer, 5′-ATTCTCAACACTCCAAACTGTGC-3′, reverse primer, 5′- ATTCTCAACACTCCAAACTGTGC-3′; and VEGF: forward primer, 5′- GAGGAGCAGTTACGGTCTGTG-3′, reverse primer, 5′-TCCTTTCCTTAGCTGACACTTGT-3′.

### Western Blot Analysis

Cells were lysed using RIPA buffer (ThermoFisher, #89900), and the cell lysate was collected, incubated on ice for 30 min, and centrifuged at 13,000 rpm for 15 min, and the supernatant was collected. A total of 10 μg of each sample was resolved by 8% SDS-PAGE, transferred to a PVDF membrane, and blocked with 5% BSA for 1 h at room temperature. Primary antibodies were added and incubated overnight at 4°C, and the secondary antibody was then incubated for one h at room temperature. Mouse monoclonal primary antibodies against rat SDF-1 (1:1000, Santa Cruz, sc-74271), VEGF (1:1000, Abcam, ab1316), GAPDH (1:1000, Abcam, ab8245), CXCR4 (1:1000, Santa Cruz, sc-53534), NRF2 (1:1000, Abcam, ab89443), and CXCR7 (1:1000, Abcam, ab72100) were used.

### CXCR7 Knockdown in EPCs

The second generation EPCs were seeded into 6-well-culture plates. When cell growth reached 80% confluence of the healing degree, NC siRNA (5′- UUCUCCGAAGUCACGU-3′), CXCR7 siRNA1 (5′- AUGAAAAUGUAGAUGAAGGAG-3′), CXCR7 siRNA2 (5′- UAGAAGAUAGCAAUGAUGGUG-3′), and CXCR7 siRNA3 (5′-UGUAAAGAGCACAUUCUCCAG-3′) were transfected into EPCs by Lipofectamine 2000 (ThermoFisher, #11668019). After 48 h of transfection, the expression of CXCR7 was detected by qPCR and Western blotting. The CXCR7 primer sequences were as follows: forward primer, 5′- CGTGCTATAGAGGCATGGGG-3′ and reverse primer, 5′- ACCACTCAAGCAACCAGACC-3′.

### Statistical Analysis

Statistical analysis was performed using Statistical Program for Social Sciences 19.0 software (SPSS, Chicago, IL, USA). Data are presented as the mean ± SD, and comparisons were analyzed by two-way ANOVA. All experiments were repeated at least three times. *P* < 0.05 was considered to indicate a statistically significant difference.

## Results

### Sitagliptin Promoted the Mobilization of EPCs to Induce Endothelialization in the Aneurysm Neck

**Figure 1:**
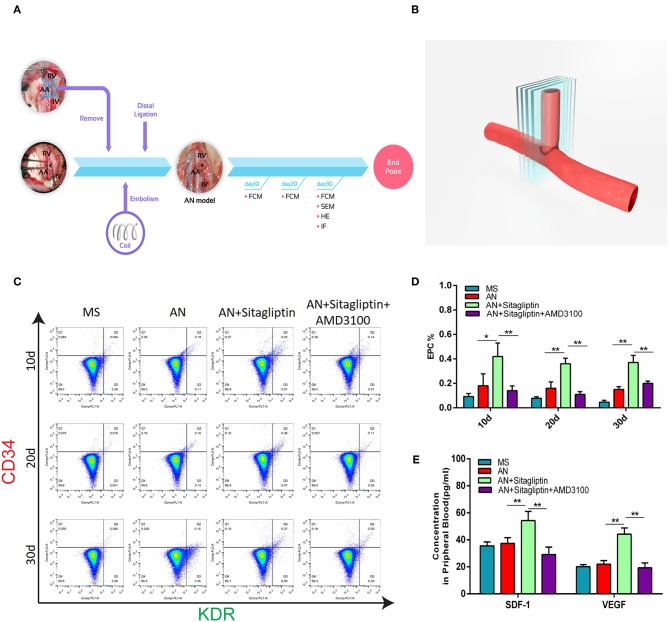
Circulating EPC detection. **(A)** Rats with aneurysms treated with coil therapy and endpoint examination of FCM, SEM, H&E, and IHC after sitagliptin treatment. **(B)** Schematic illustration showing the coiled aneurysm tissue embedded and then cut into sections (5-μm thick) perpendicular to the long axis of the parent vessel. Circulating EPCs were counted **(C)**, and quantitative analysis **(D)** was performed by FCM on days 10, 20, and 30 in MS rats, untreated AN rats, AN+Sitagliptin rats (via gavage in normal saline at 15 mg/kg/day every day during treatment) and AN+Sitagliptin+AMD3100 (intraperitoneally in normal saline at 1.25 mg/kg/day combined with Sitagliptin via gavage every day during treatment) rats. **(E)** The concentration of SDF-1 and VEGF in peripheral blood of MS rats, untreated AN rats, AN+Sitagliptin rats, and AN+Sitagliptin+AMD3100 rats. All experiments were performed three independent times, and representative images are shown. Data are expressed as the mean ± SD; ^*^P < 0.05 and ^**^P < 0.01 compared to Blank.

**Figure 2:**
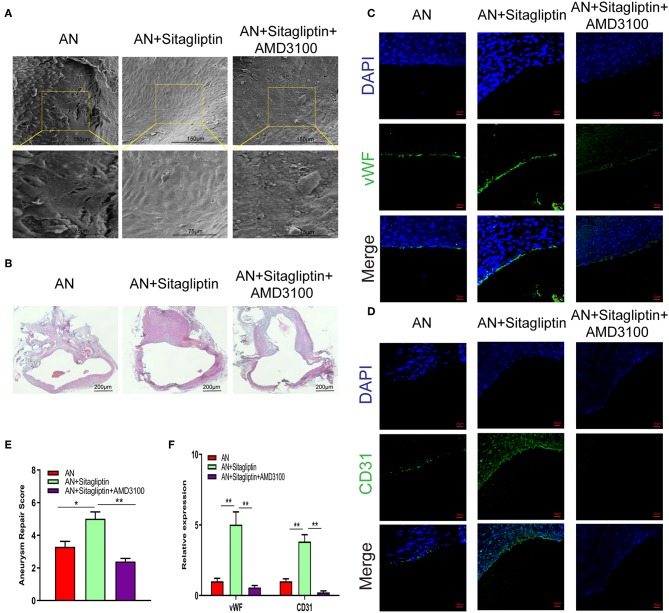
SEM observation and immunofluorescence. Photomicrographs showing the endothelialization in the AN neck under SEM **(A)** (scale bar: 75 μm) and with H&E staining **(B)** (scale bar: 200 μm). Immunofluorescence detection of endothelial cells by vWF **(C)** (scale bar: 20 μm) and CD31 **(D)** (scale bar: 20 μm) and quantitative analysis **(E,F)**. All experiments were performed three independent times, and representative images are shown. Data are expressed as the mean ± SD; ^*^P < 0.05 and ^**^P < 0.01 compared to Blank; *n* = 6.

According to previous studies, sitagliptin increases circulating levels of SDF-1, which has an important role in inducing endothelialization in aneurysm necks (21), and so we investigated the role of sitagliptin in endothelialization and mobilization of EPCs. The results showed that sitagliptin significantly increased the number of EPCs in the peripheral blood, while AMD3100 (a symmetrical bicyclam compound that antagonizes SDF-1 binding to CXCR4) significantly decreased the level of EPCs and VEGF in the peripheral blood (Figures 1A–E). Furthermore, scanning electron microscopy (Figure 2A), H&E staining (Figure 2B) and immunofluorescence detection of vWF (Figure 2C) and CD31 (Figure 2D) in the necks of aneurysms showed that sitagliptin significantly promoted endothelialization of the aneurysmal neck, while AMD3100 inhibited endothelialization of the aneurysmal neck (Figures 2E,F). These results showed that sitagliptin promoted the mobilization of EPCs to induce endothelialization in the aneurysmal neck through the SDF-1/CXCR4 signaling pathway.

### Sitagliptin Promoted the Proliferation of EPCs

**Figure 3:**
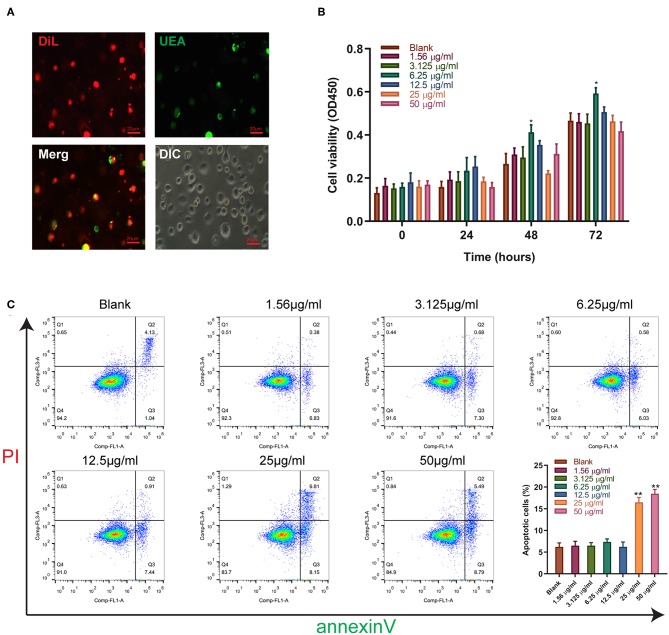
The effect of sitagliptin on the viability of EPCs. **(A)** EPCs were identified by Dil and UEA staining (scale bar: 20 μm). **(B)** CCK-8 assays were performed with varying doses of sitagliptin for 24, 48, and 72 h. **(C)** FCM and quantitative analysis of the apoptotic rate of EPCs treated with varying doses of sitagliptin for 24 h. All experiments were performed three independent times, and representative images are shown. Data are expressed as the mean ± SD; ^*^P < 0.05 and ^**^P < 0.01 compared to Blank.

We further validated the effect of sitagliptin on EPCs. To verify that the cells we isolated were EPCs, we performed immunofluorescence assays that showed high expression of Dil and UEA (Figure 3A). The effect of sitagliptin on the proliferation of EPCs was examined by a CCK-8 assay. The results showed that 6.25 μg/ml sitagliptin significantly promoted the proliferation of EPCs (Figure 3B). Flow cytometry analysis showed that sitagliptin did not cause apoptosis of EPCs at concentrations below 6.25 μg/ml, and EPC apoptosis increased significantly at concentrations of 25 and 50 μg/ml (Figure 3C).

### Sitagliptin Promoted the Migration and Angiogenesis of EPCs

**Figure 4:**
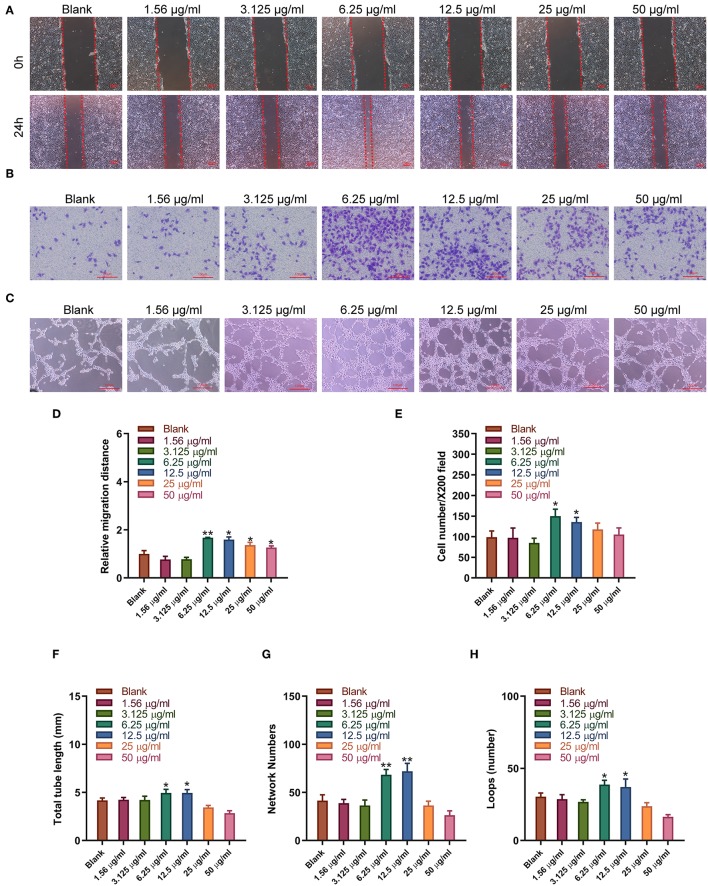
Sitagliptin promoted the migration and angiogenesis of EPCs. **(A)** Wound healing assays (scale bar: 200 μm) and quantitative analysis **(D)** were performed to determine the migration ability of EPCs with varying doses of sitagliptin for 24 h. **(B)** Transwell assays (scale bar: 100 μm) and quantitative analysis **(E)** were performed to determine the migration ability of EPCs with varying doses of sitagliptin for 24 h. **(C)** An angiogenesis assay (scale bar: 100 μm) and quantitative analysis **(F–H)** were performed to determine the angiogenesis ability of EPCs with varying doses of sitagliptin for 24 h. All experiments were performed three independent times, and representative images are shown. Data are expressed as the mean ± SD; ^*^P < 0.05 and ^**^P < 0.01 compared to Blank.

Sitagliptin was observed to significantly promote the migration of EPCs, and sitagliptin promoted EPC migration most significantly at a concentration of 6.25 μg/ml (Figure 4A). To further examine the effect of sitagliptin on the invasion of EPCs, we performed a Transwell assay that showed that sitagliptin significantly promoted the invasion of EPCs (Figure 4B). EPCs play a crucial role in the formation of blood vessels. Therefore, we further examined the effects of sitagliptin on the angiogenic ability of EPCs. The results of the angiogenesis experiments showed that sitagliptin significantly promoted vascularization at concentrations of 6.25 and 12.5 μg/ml (Figure 4C). Quantitative analysis of wound healing (Figure 4D), Transwell assays (Figure 4E), and angiogenesis assays (Figures 4F–H) with varying doses of sitagliptin for 24 h showed the same results.

### Sitagliptin Promoted the Expression of SDF-1 and VEGF in EPCs

**Figure 5:**
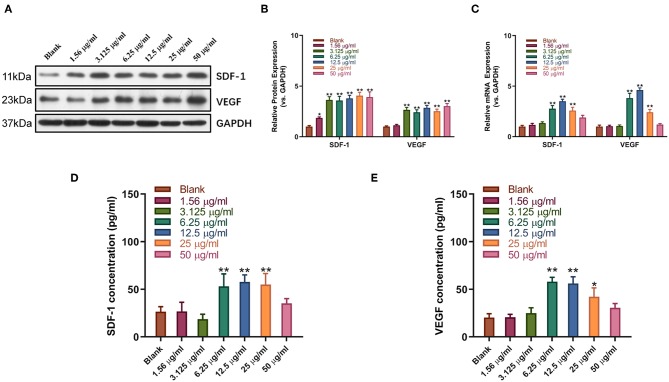
Sitagliptin promoted the expression of SDF-1 and VEGF in EPCs. Western blot assays **(A)** and quantitative analysis **(B)** were conducted to determine SDF-1 and VEGF protein expression in EPCs with varying doses of sitagliptin for 48 h. **(C)** qPCR was performed to determine SDF-1 and VEGF mRNA expression in EPCs with varying doses of sitagliptin for 48 h. ELISA assays were performed to determine the protein expression of SDF-1 **(D)** and VEGF **(E)** in EPC supernatants with varying doses of sitagliptin for 48 h. All experiments were performed three independent times, and representative images are shown. Data are expressed as the mean ± SD; ^*^P < 0.05 and ^**^P < 0.01 compared to Blank.

Western blot and qPCR analysis showed that sitagliptin significantly promoted the expression of VEGF and SDF-1 at the protein and mRNA levels (Figures 5A–C). ELISA showed that sitagliptin promoted the expression of VEGF and SDF-1 in culture supernatants of EPCs at 6.25, 12.5, and 25 μg/ml (Figures 5D,E).

### Sitagliptin Promoted the Migration and Angiogenesis of EPCs by the SDF-1/CXCR4 Signaling Pathway

**Figure 6:**
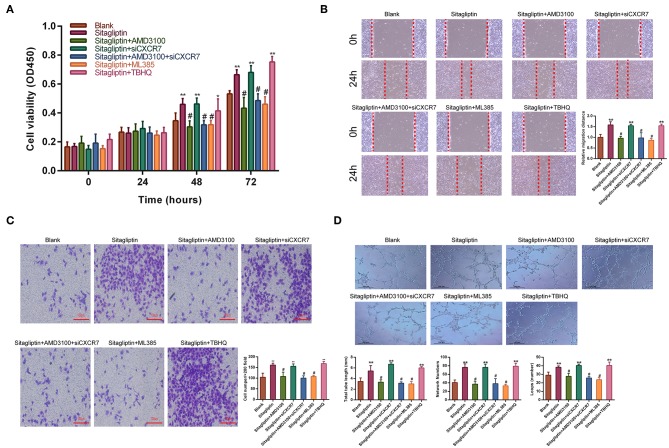
AMD3100 inhibited the effects of sitagliptin on EPCs. **(A)** CCK-8 assays were performed to determine the proliferation ability of EPCs with sitagliptin and a CXCR4 inhibitor, CXCR7 siRNA and a NRF2 inhibitor or agonist (TBHQ). **(B)** Wound healing assays and quantitative analyses were performed to determine the migration ability of EPCs with sitagliptin and a CXCR4 inhibitor (AMD3100, 50 nM), CXCR7 siRNA and a NRF2 inhibitor (ML385, 5 μM) or agonist (TBHQ, 5 μM). (scale bar: 100 μm). **(C)** Transwell assays and quantitative analyses were performed to determine the migration ability of EPCs with sitagliptin and a CXCR4 inhibitor, CXCR7 siRNA and a NRF2 inhibitor (ML385) or agonist (TBHQ) (scale bar: 100 μm). **(D)** An angiogenesis assay and quantitative analysis were performed to determine the angiogenesis ability of EPCs with sitagliptin and a CXCR4 inhibitor, CXCR7 siRNA and a NRF2 inhibitor (ML385) or agonist (TBHQ) (scale bar: 100 μm). All experiments were performed three independent times, and representative images are shown. Data are expressed as the mean ± SD; ^*^P < 0.05 and ^**^P < 0.01 compared to Blank; ^#^*P* < 0.05 compared to sitagliptin.

Sitagliptin prevents SDF-1, which has two receptors, CXCR4 and CXCR7, from degradation by inhibiting DPP-IV. Therefore, we used AMD3100 (a CXCR4-specific antagonist) and CXCR7 siRNA (Figure S1) to analyze whether the promotion of EPC migration and angiogenesis by sitagliptin occurs through the SDF-1/CXCR4 or SDF-1/CXCR7 signaling pathway. The results showed that after the addition of AMD3100, the effect of sitagliptin on the proliferation, migration, and invasion of EPCs was significantly reduced (Figures 6A–C). AMD3100 also significantly reduced the promoting effect of sitagliptin on EPC angiogenesis (Figure 6D); however, CXCR7 knockdown did not reduce the effect of sitagliptin on EPCs. To further verify whether Nrf2 is involved in the regulation of endothelial progenitor cells, the NRF2 inhibitor ML385 (Sigma-Aldrich, #SML1833) (inhibits the activity of the Nrf2 transcription factor by binding to Neh1, a CNC-bZIP domain that allows Nrf2 to heterodimerize with small Maf proteins, blocking NRF2 transcriptional activity) was used and decreased the effect of sitagliptin on EPCs, while the NRF2 agonist (TBHQ is a widely used Nrf2 activator) promoted the proliferation, migration, invasion, and angiogenesis of EPCs.

### Sitagliptin Promoted the Expression of CXCR4 and NRF2 in EPCs

**Figure 7:**
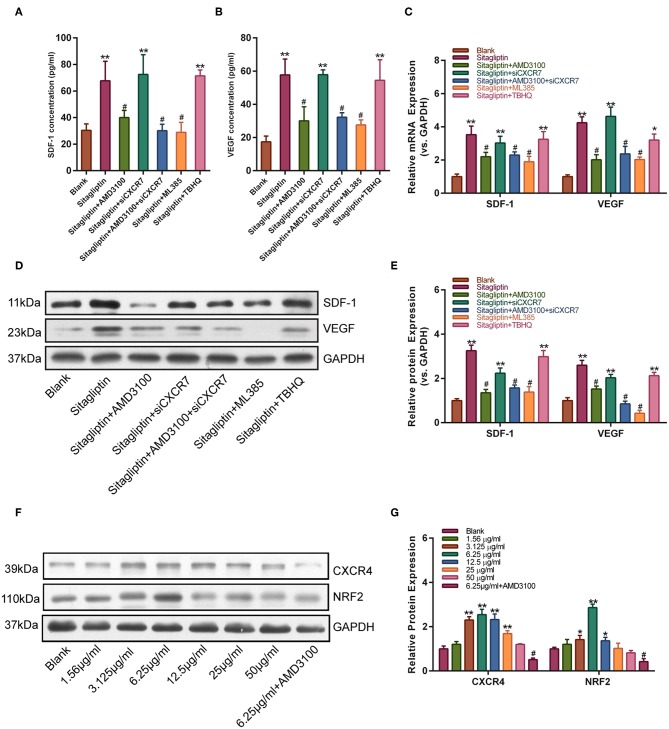
AMD3100 inhibited the sitagliptin-induced expression of SDF-1 and VEGF in EPCs. ELISA was performed to determine SDF-1 **(A)** and VEGF **(B)** protein expression in EPC supernatants with sitagliptin and a CXCR4 inhibitor, CXCR7 siRNA and a NRF2 inhibitor (ML385) or agonist (TBHQ) for 48 h. **(C)** qPCR was performed to determine SDF-1 and VEGF mRNA expression in EPCs with sitagliptin and a CXCR4 inhibitor, CXCR7 siRNA and a NRF2 inhibitor (ML385) or agonist (TBHQ) for 48 h. **(D)** Western blot assays and **(E)** quantitative analysis were performed to determine the protein expression of SDF-1 and VEGF in EPCs with sitagliptin and a CXCR4 inhibitor (AMD3100), CXCR7 siRNA and a NRF2 inhibitor (ML385) or agonist (TBHQ) for 48 h. **(F)** Western blot assays and **(G)** quantitative analysis were performed to determine the protein expression of CXCR4 and NRF2 in EPCs with different concentrations of sitagliptin alone or in combination with AMD3100. All experiments were performed three independent times, and representative images are shown. Data are expressed as the mean ± SD; ^*^P < 0.05 and ^**^P < 0.01 compared to Blank; ^#^*P* < 0.05 compared to sitagliptin.

We further tested the effect of AMD3100 on the expression of SDF-1 and VEGF. Western blot analysis showed that the sitagliptin-induced expression of SDF-1 and VEGF was significantly inhibited by AMD3100 and the NRF2 inhibitor (Figures 7A,B). qPCR analysis also showed similar results (Figure 7C). The expression of SDF-1 and VEGF in EPC culture supernatants was analyzed by ELISA. The results showed that AMD3100 significantly reduced the secretion of SDF-1 and VEGF by cells treated with sitagliptin (Figures 7D–F). Western blot analysis showed that sitagliptin significantly promoted the expression of CXCR4 and NRF2, while AMD3100 significantly inhibited the expression of CXCR4 and NRF2 (Figure 7G).

## Discussion

In this study, we established a model of rat aneurysm embolization to study the effect of sitagliptin on aneurysm neck endothelialization. Although there are some differences between peripheral blood vessels and intracranial blood vessels, the goal of our study was mainly to examine the process of endothelialization of the aneurysmal neck after aneurysm embolization, rather than the process of aneurysm itself. Therefore, for the process of aneurysmal neck endothelialization, we believe that the results of this model can be extended to the aneurysm after intracranial embolization, and an improved model of vascular embolization is the direction of our future research. In this study, we observed that sitagliptin increased the number of endothelial cells in the peripheral blood of the rat aneurysm model. Moreover, it was observed that the degree of endothelialization of the aneurysmal neck in the treatment group increased significantly.

The protective effect of sitagliptin on endothelial progenitor cell function has been a popular topic this year. Sitagliptin may protect vascular endothelial cells by inhibiting the degradation of SDF-1. SDF-1 is a chemokine that has a strong chemotactic effect on hematopoietic stem/endothelial cells. After vascular injury, a high SDF-1 concentration gradient can form locally, thereby inducing endothelial progenitor cells to mobilize from the bone marrow along the concentration gradient to the periphery, further homing to the vascular injury area, and promoting endothelialization repair through adhesion, angiogenesis, and paracrine signals (22, 23). Our study showed that sitagliptin promoted not only the migration and angiogenesis of endothelial progenitor cells but also the expression of the provascular cytokines SDF-1 and VEGF by endothelial progenitor cells at the mRNA and protein levels. Previous studies have shown that SDF-1 works through its unique receptor CXCR4 (24, 25), but it also has a completely independent receptor, CXCR7 (26, 27), which has a different role from CXCR4. To determine which SDF-1 receptor mediates the effects of sitagliptin, after screening for the optimal drug concentration of sitagliptin, we used treated endothelial progenitor cells with sitagliptin *in vitro* while adding a CXCR4 antagonist or CXCR7 siRNA to block CXCR4 or silence CXCR7 expression, respectively. The results showed that after the addition of the CXCR4 antagonist, the angiogenic function of endothelial progenitor cells was significantly inhibited, and the promotion of SDF-1 and VEGF expression was also significantly reduced; however, after CXCR7 expression was silenced, the effect of sitagliptin on the function of endothelial progenitors was not affected. Therefore, we believe that sitagliptin regulates the function of endothelial progenitor cells through the SDF-1/CXCR4 signaling pathway. To prove this, we used an animal model of aneurysm embolization to verify this effect *in vivo*. We successfully verified the pro-endothelialization effect of sitagliptin on the neck of an embolized aneurysm. We divided the rats into four groups, two of which were treated with sitagliptin with or without the CXCR4 receptor antagonist AMD3100 (28, 29). The results showed that sitagliptin significantly increased the number of EPCs in the peripheral blood. The histology and scanning electron microscopy results showed that sitagliptin strongly increased the endothelialization of the aneurysm after embolization, and this enhancement was blocked by the CXCR4 antagonist AMD3100. Immunofluorescence assays showed that sitagliptin significantly promoted the expression of vWF and CD31 in endothelial cells in the aneurysmal necks, which was blocked by AMD3100. Therefore, we hypothesize that sitagliptin regulates endothelialization through the CXCR4 signaling pathway.

A previous study by our research team found that NRF2 plays an important role in the development of rat aneurysms (30). Therefore, we further studied whether NRF2 is involved in the effect of sitagliptin on the endothelialization of aneurysms. In this study, we added the NRF2 antagonist ML385 and agonist TBHQ *in vitro*, individually and in combination with sitagliptin. The angiogenic function of endothelial progenitor cells and the ability to secrete SDF-1 and VEGF were also inhibited after the addition of an NRF2 antagonist. We believe that NRF2, as a target protein downstream of SDF-1, is involved in the process of promoting the endothelialization of endothelial progenitor cells, which may be attributed to the strong antioxidant resistance of NRF2. We intend to further verify this *in vivo*. However, the origin of endothelial cells in the aneurysmal neck is not clear. This is also a further problem for our team to investigate.

## Conclusions

**Figure 8:**
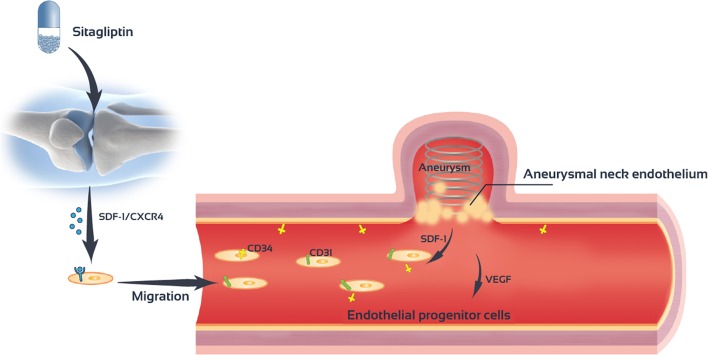
Sitagliptin stimulates endothelial progenitor cells to induce endothelialization in the aneurysmal neck through the SDF-1/CXCR4/NRF2 signaling pathway.

Sitagliptin promotes the mobilization of EPCs cells and increase the number of EPCs in the peripheral blood. Sitagliptin also promotes the migration and angiogenesis of EPCs. The SDF-1/CXCR4 signaling pathway mediates the role of sitagliptin in EPC mobilization and angiogenesis (Figure 8).

## Data Availability Statement

The raw data supporting the conclusions of this manuscript will be made available by the authors, without undue reservation, to any qualified researcher.

## Ethics Statement

This animal study was reviewed and approved by Fudan University.

## Author Contributions

GY and WZ designed all experiments. GY, PL, and YS experiments. GY and SL collected and analyzed data. GY and YL drafted this manuscript. All authors read and approved this manuscript.

### Conflict of Interest

The authors declare that the research was conducted in the absence of any commercial or financial relationships that could be construed as a potential conflict of interest.

## Supplementary Material

Figure S1 CXCR7 expression was suppressed by siRNA. **(A)** qPCR was performed to determine CXCR7 mRNA expression in EPCs transfected with NC siRNA or CXCR7 siRNA. **(B)** Western blotting was performed to determine CXCR7 protein expression in EPCs transfected with NC siRNA or CXCR7 siRNA. All experiments were performed three independent times, and representative images are shown. Data are expressed as the mean ± SD; ^**^*P* < 0.01 compared to NC siRNA.

## References

[1] Frosen J Tulamo R Paetau A Laaksamo E Korja M Laakso A. Saccular intracranial aneurysm: pathology and mechanisms. Acta Neuropathol. (2012) 123:773–86. 10.1007/s00401-011-0939-3 22249619

[2] Frosen J Cebral J Robertson AM Aoki T. Flow-induced, inflammation-mediated arterial wall remodeling in the formation and progression of intracranial aneurysms. Neurosur Focus. (2019) 47:E21. 10.3171/2019.5.FOCUS19234 31261126 PMC7193287

[3] Xu Z Rui YN Hagan JP Kim DH. Intracranial aneurysms: pathology, genetics, and molecular mechanisms. Neuromolecular Med. (2019) 7:1–19. 10.1007/s12017-019-08537-7 31055715 PMC6829066

[4] Liu P An Q Chen X Huang J Yang GY Zhu W. Rosuvastatin for enhancement of aneurysm neck endothelialization after coil embolization: promotion of endothelial progenitor cells in a rodent model. J neurosurg. (2016) 124:1265–74. 10.3171/2015.3.JNS142841 26406802

[5] Raymond J Darsaut T Salazkin I Gevry G Bouzeghrane F. Mechanisms of occlusion and recanalization in canine carotid bifurcation aneurysms embolized with platinum coils: an alternative concept. AJNR. Am J Neuroradiol. (2008) 29:745–52. 10.3174/ajnr.A0902 18202238 PMC7978218

[6] Liu P Zhou Y An Q Song Y Chen X Yang GY. Erythropoietin stimulates endothelial progenitor cells to induce endothelialization in an aneurysm neck after coil embolization by modulating vascular endothelial growth factor. Stem Cells Transl Med. (2016) 5:1182–9. 10.5966/sctm.2015-0264 27352930 PMC4996438

[7] Asahara T Murohara T Sullivan A Silver M van der Zee R Li T. Isolation of putative progenitor endothelial cells for angiogenesis. Science. (1997) 275:964–7. 10.1126/science.275.5302.964 9020076

[8] Liu F Liu ZD Wu N Wang JH Zhang HH Fei R. *In vitro* interactions between rat bone marrow-derived endothelial progenitor cells and hepatic stellate cells: interaction between EPCs and HSCs. In Vitro Cell Dev Biol Anim. (2013) 49:537–47. 10.1007/s11626-013-9637-x 23722413

[9] Goligorsky MS, Salven P. Concise review: endothelial stem and progenitor cells and their habitats. Stem Cells Transl Med. (2013)2:499–504. 10.5966/sctm.2013-0005 23761107 PMC3697817

[10] Schatteman GC Dunnwald M Jiao C. Biology of bone marrow-derived endothelial cell precursors. Am J Physiol. Heart Circ Physiol. (2007) 292:H1–18. 10.1152/ajpheart.00662.2006 16980351

[11] Jamous MA Nagahiro S Kitazato KT Satoh K Satomi J. Vascular corrosion casts mirroring early morphological changes that lead to the formation of saccular cerebral aneurysm: an experimental study in rats. J Neurosurg. (2005) 102:532–5. 10.3171/jns.2005.102.3.0532 15796390

[12] Wuchter P Leinweber C Saffrich R Hanke M Eckstein V Ho AD. Plerixafor induces the rapid and transient release of stromal cell-derived factor-1 alpha from human mesenchymal stromal cells and influences the migration behavior of human hematopoietic progenitor cells. Cell Tissue Res. (2014) 355:315–26. 10.1007/s00441-013-1759-7 24337688

[13] Masyuk M Abduelmula A Morosan-Puopolo G Ödemis V Rehimi R Khalida N. Retrograde migration of pectoral girdle muscle precursors depends on CXCR4/SDF-1 signaling. Histochem Cell Biol. (2014) 142:473–88. 10.1007/s00418-014-1237-7 24972797

[14] Hattori K. Heissig B Tashiro K Honjo T Tateno M Shieh JH. Plasma elevation of stromal cell-derived factor-1 induces mobilization of mature and immature hematopoietic progenitor and stem cells. Blood. (2001) 97:3354–60. 10.1182/blood.V97.11.3354 11369624

[15] Hu TH Yao Y Yu S Han LL Wang WJ Guo H. SDF-1/CXCR4 promotes epithelial-mesenchymal transition and progression of colorectal cancer by activation of the Wnt/beta-catenin signaling pathway. Cancer Lett. (2014) 354:417–26. 10.1016/j.canlet.2014.08.012 25150783

[16] Moi P Chan K Asunis I Cao A Kan YW. Isolation of NF-E2-related factor 2 (Nrf2), a NF-E2-like basic leucine zipper transcriptional activator that binds to the tandem NF-E2/AP1 repeat of the beta-globin locus control region. Proc Natl Acad Sci USA. (1994) 91:9926–30. 10.1073/pnas.91.21.9926 7937919 PMC44930

[17] Tsai JJ Dudakov JA Takahashi K Shieh JH Velardi E Holland AM. Nrf2 regulates haematopoietic stem cell function. Nat Cell Biol. (2013) 15:309–16. 10.1038/ncb2699 23434824 PMC3699879

[18] Green JB Bethel MA Armstrong PW Buse JB Engel SS Garg J. Effect of Sitagliptin on Cardiovascular Outcomes in Type 2 Diabetes. N Engl J Med. (2015) 373:232–42. 10.1056/NEJMoa1501352 26052984

[19] Remm F Kränkel N Lener D Drucker DJ Sopper S Brenner C. Sitagliptin accelerates endothelial regeneration after vascular injury independent from GLP1 receptor signaling. Stem Cells Int. (2018) 2018:5284963. 10.1155/2018/5284963 29531541 PMC5822806

[20] Nade VS Kawale LA Patel KM. Protective effect of sitagliptin and rosuvastatin combination on vascular endothelial dysfunction in type-2 diabetes. Indian J Pharm Sci. (2015) 77:96–102. 10.4103/0250-474X.151604 25767324 PMC4355889

[21] Papazafiropoulou AK Papanas N Trikkalinou A Fousteris E Melidonis A. The oral dipeptidyl-peptidase-4 inhibitor sitagliptin increases circulating levels of stromal-derived factor-1 alpha. Exp Clin Endocrinol Diabetes. (2018) 126:367–70. 10.1055/s-0043-118748 28931178

[22] Piao JM Wu W Yang ZX Li YZ Luo Q Yu JL. MicroRNA-381 favors repair of nerve injury through regulation of the SDF-1/CXCR4 signaling pathway via LRRC4 in Acute cerebral ischemia after cerebral lymphatic blockage. Cell Physiol Biochem. (2018) 46:890–906. 10.1159/000488821 29669322

[23] Thomas MN. Kalnins A Andrassy M Wagner A Klussmann S Rentsch M. SDF-1/CXCR4/CXCR7 is pivotal for vascular smooth muscle cell proliferation and chronic allograft vasculopathy. Transpl Int. (2015) 28:1426–35. 10.1111/tri.12651 26265085

[24] Zhou Y Cao HB Li WJ Zhao L. The CXCL12 (SDF-1)/CXCR4 chemokine axis: oncogenic properties, molecular targeting, and synthetic and natural product CXCR4 inhibitors for cancer therapy. Chin J Nat Med. (2018) 16:801–10. 10.1016/S1875-5364(18)30122-5 30502762

[25] Mirandola L Apicella L Colombo M Yu Y Berta DG Platonova N. Anti-notch treatment prevents multiple myeloma cells localization to the bone marrow via the chemokine system CXCR4/SDF-1. Leukemia. (2013) 27:1558–66. 10.1038/leu.2013.27 23354012

[26] Burns JM Summers BC Wang Y Melikian A Berahovich R Miao Z. A novel chemokine receptor for SDF-1 and I-TAC involved in cell survival, cell adhesion, and tumor development. J Exp Med. (2006) 203:2201–13. 10.1084/jem.20052144 16940167 PMC2118398

[27] Tarnowski M Liu R Wysoczynski M Ratajczak J Kucia M Ratajczak MZ. CXCR7: a new SDF-1-binding receptor in contrast to normal CD34(+) progenitors is functional and is expressed at higher level in human malignant hematopoietic cells. Eur J Haematol. (2010) 85:472–83. 10.1111/j.1600-0609.2010.01531.x 20887389

[28] Reeves PM Abbaslou MA Kools FRW Poznansky MC. CXCR4 blockade with AMD3100 enhances Taxol chemotherapy to limit ovarian cancer cell growth. Anti Cancer Drugs. (2017) 28:935–42. 10.1097/CAD.0000000000000518 28817386

[29] Liu JM Zhao K Du LX Zhou Y Long XH Chen XY. AMD3100 inhibits the migration and differentiation of neural stem cells after spinal cord injury. Sci Rep. (2017) 7:64. 10.1038/s41598-017-00141-8 28246405 PMC5427924

[30] Shi Y Li S Song Y Liu P Yang Z Liu Y. Nrf-2 signaling inhibits intracranial aneurysm formation and progression by modulating vascular smooth muscle cell phenotype and function. J Neuroinflammation. (2019) 16:185. 10.1186/s12974-019-1568-3 31585542 PMC6778377

