# Supplementary figures and images for: Sitagliptin Stimulates Endothelial Progenitor Cells to Induce Endothelialization in Aneurysm Necks Through the SDF-1/CXCR4/NRF2 Signaling Pathway
Source: Front Endocrinol (Lausanne). 2019 Nov 26;10:823. doi: 10.3389/fendo.2019.00823 (PMC6988800; doi:10.3389/fendo.2019.00823)

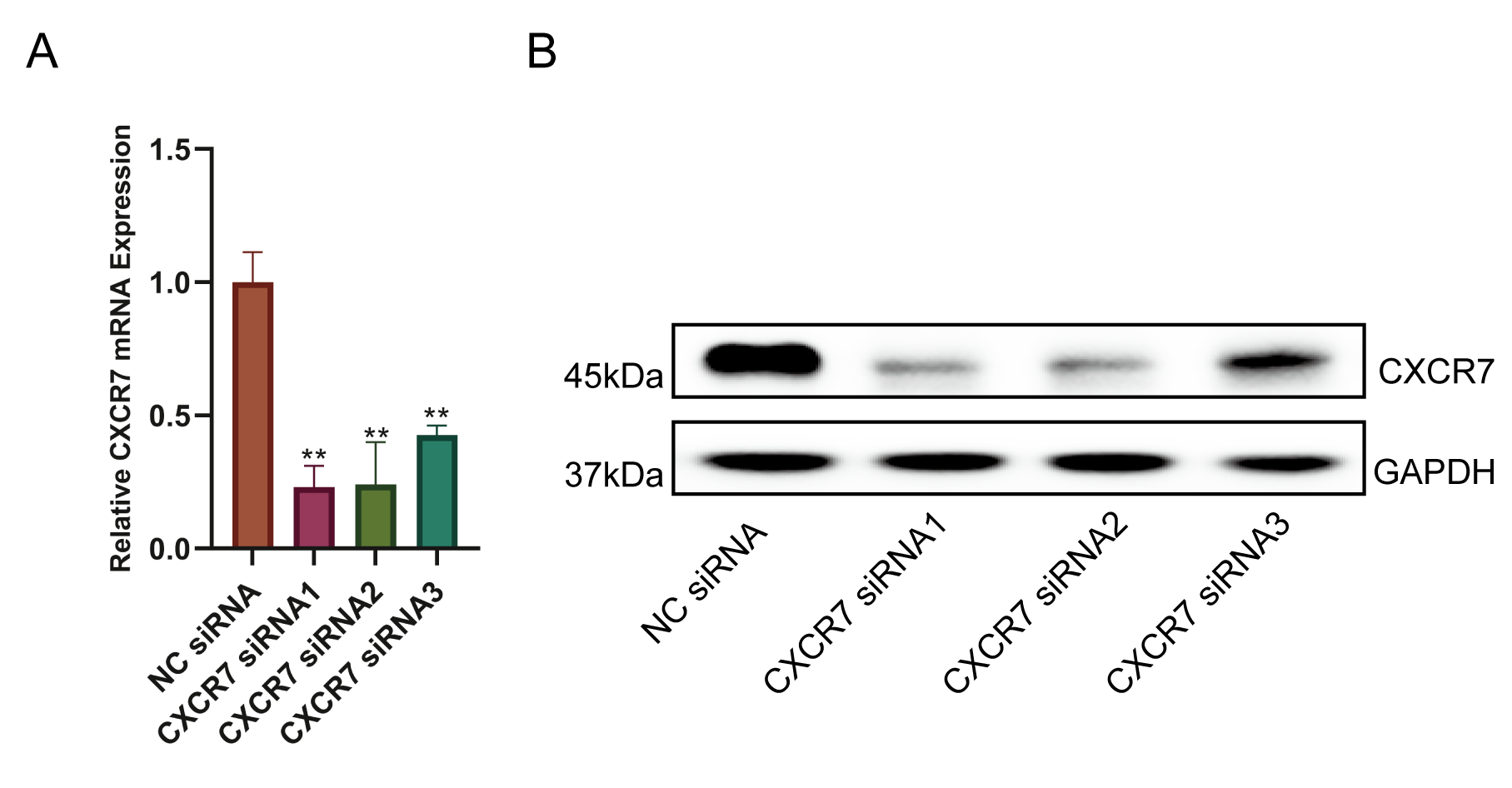

Supplement: Figure S1 — CXCR7 expression was suppressed by siRNA. (A) qPCR was performed to determine CXCR7 mRNA expression in EPCs transfected with NC siRNA or CXCR7 siRNA. (B) Western blotting was performed to determine CXCR7 protein expression in EPCs transfected with NC siRNA or CXCR7 siRNA. All experiments were performed three independent times, and representative images are shown. Data are expressed as the mean ± SD; **P < 0.01 compared to NC siRNA. [file Image_1.TIF]
